# A Prospective Study of Medication Surveillance of a Pediatric Tertiary Care Hospital in Lahore, Pakistan

**DOI:** 10.3390/pediatric14020038

**Published:** 2022-06-15

**Authors:** Hafiz Awais Nawaz, Tahir Mehmood Khan, Qendeel Adil, Khang Wen Goh, Long Chiau Ming, Ali Qais Blebil, Kah Seng Lee, Jagjit Singh Dhaliwal

**Affiliations:** 1 Institute of Pharmaceutical Sciences, University of Veterinary & Animal Sciences, Lahore 54000, Pakistan; tahir.khan@uvas.edu.pk (T.M.K.); qandeeladil@gmail.com (Q.A.); 2 School of Pharmacy, Monash University Malaysia, Bandar Sunway 47500, Malaysia; ali.blebil@monash.edu; 3 Faculty of Data Science and Information Technology, INTI International University, Nilai 71800, Malaysia; 4 PAPRSB Institute of Health Sciences, Universiti Brunei Darussalam, Gadong BE1410, Brunei; longchiauming@gmail.com (L.C.M.); jagjit.dhaliwal@ubd.edu.bn (J.S.D.); 5 Faculty of Pharmacy, University of Cyberjaya, Cyberjaya 63000, Malaysia; ksl.pharm@gmail.com; 6 Faculty of Dentistry, University of Health Sciences, Lahore 54600, Pakistan

**Keywords:** drug safety, drug-related problems, drug-drug interaction, clinical pharmacy

## Abstract

Purpose: Several studies have shown that polypharmacy is the main cause of drug interactions, and the prevalence and the level of the severity varied with the duration of stay in the hospital, sex and race of the patients. The aims of this investigation were to identify the drug-drug interactions in hospitalized pediatric patients associated with polypharmacy, and to categorize the drug interactions in pharmacokinetic or pharmacodynamic interactions according to their level of severity. Methods: A cross-sectional, prospective analytical study was performed at a pediatric tertiary care hospital in Lahore, Pakistan for the duration of 4 months, which included prescription orders for 300 patients. Data were collected from patient medical files about previous and current medication history. Drug interactions were analyzed using interaction checker on Medscape and categorized according to the severity levels. Results: Out of 300 patients, the occurrence of drug interactions was found in 157 (52.3%) patients, while in 143 (47.7%), no interaction was found. Among these interactions, 50.7% were pharmacodynamic interactions, and 49.30% were pharmacokinetic interactions. Eighty-one percent of prescription orders with drug interactions contained more than three drugs, and 11.9% of interactions were severe. The majority of interactions were of amikacin-vancomycin, piroxicam-captopril and captopril-ciprofloxacin. Conclusion: Most of the interactions were moderate among patients with multiple drug prescriptions. The drug interactions can be minimized by providing special patient monitoring and adequate management with prior knowledge of these drug interaction.

## 1. Introduction

Polypharmacy is one of the major reasons and the foremost vulnerability factor for drug-drug interactions (DDIs) in the tertiary health care setting [1]. There is no universal consensus on the definition of polypharmacy [2,3], but many researchers describe it as the concurrent use of 5 or more medications in a single prescription order [3,4]. Reasons for polypharmacy include multiple comorbidities, receiving advice from several physicians for the same disease at the same time, hospitalization and lack of education among the patients [5]. In any case, it leads to enhanced drug costs and compromised patient quality of life, but the most worrisome is the increased risk of adverse effects in patients due to the interaction of some of these co-prescribed medicines [5,6]. Decreasing the number of drugs per prescription can effectively lessen the adverse effects based on DDIs. However, sometimes in chronically ill patients with multiple comorbidities, polypharmacy cannot be avoided. Drug interactions, arising due to polypharmacy, can be minimized or managed when the prescribers and pharmacists have proper knowledge of action approach, pharmacogenomics, pharmacokinetics including absorption, elimination and metabolism of drugs as well as the clinical expertise to predict the risk factors. For instance, phenytoin is a drug inducer of CYP3A4 enzyme, which is responsible for metabolism of gefitinib [7]. As a result, its plasma concentration and pharmacological activity are reduced. Such types of interactions can be avoided by using alternative treatment options, dosage adjustments and spacing the time of administration of interacting drugs [6].

Generally, patients who experience adverse drug reactions (ADRs), have been taking many medicines simultaneously and have prolonged hospitalization. The chances of occurrence of an ADR episode have been reported to multiply by 1.14% with every additional medicine in the prescription [8]. Information regarding the occurrence of adverse drug reactions, prevention of medication errors and epidemiology in pediatric inpatient settings is scarce [9]. Health systems face distinct challenges in pediatric patients in terms of prescribing, dispensing, administering and monitoring medications. For example, as most of the pediatric dose calculations are based on patient’s body weight, prescribing medications generally requires rigorous calculations as compared to normal adults. Chances of error are also high as dispensing the pediatric medicine often requires dilution of stock solutions. Inability of younger patients to communicate with clinicians regarding possible mistakes in medicine administration could also be a problem in this regard. Several studies showed that polypharmacy was the main cause of drug interactions, and the prevalence and level of severity varied according to the duration of hospitalization, sex and race of the patients [9,10,11,12]. A study from Pakistan has reported that the prevalence of drug interactions was higher in cardiac patients and was dependent on age, gender and other diseases [13].

Most of the conducted studies regarding drug related problems are on adult patients, but pediatric and geriatric patients are at high risk of drug interactions because of compromised physiology and comorbidities. Thus, this study aimed to identify drug-drug interactions in hospitalized pediatric patients associated with polypharmacy and to categorize the drug interactions in pharmacokinetic (kinetics of drugs within the body) or pharmacodynamic (actions of drugs on the body) interactions according to their level of severity.

## 2. Methods

### 2.1. Study Design and Setting

This was a cross-sectional, prospective study carried out at one public pediatric tertiary care hospital in Lahore, Pakistan for the duration of four months, from 1 September to 31 December 2019. A structured data collection tool was finalized in a review of published literature by the research team. It comprised numbered items, and these items were shortlisted after consultation with the senior physicians practicing within the hospital. Moreover, any other information that was considered essential during data collection was noted regardless of the presence of this item in the data collection tool. Keeping in view the study objectives, an observational study design was recommended in the literature. A majority of studies investigating similar data have adopted a cross-sectional study design. In addition to allowing making solid recommendations, prospective data collection is preferred over retrospective data. The data were collected by reviewing the previous and current medication histories based on medical records of hospitalized pediatric patients who were admitted in different wards of the hospital. Drug interactions were analyzed by using the interaction checker on ‘Medscape drug interaction checker’, easily available online and accredited by the Accreditation Council for Continuing Medical Education (ACCME, Chicago IL, United States) (https://reference.medscape.com/drug-interactionchecker (accessed on 10 September 2021))and categorized according to severity level, i.e., severe, moderate and minor, as described by Medscape (https://reference.medscape.com/drug-interactionchecker (10 September 2022))or other research [14]. Samples were selected using a random number generator in Excel (Microsoft Office Professional Plus 2016). The inclusion criterion was in-patients undergoing treatment at different wards of the hospital. The maximum age limit was 14 years. None of the patients undergoing critical surgery or admitted to the intensive care unit (ICU) were included in the study. As all of the patients were under 18, their guardians were guided through verbal and written literature and consent was obtained. A patient was excluded from the study if their guardian withdrew consent at any time during the study.

### 2.2. Ethical Consideration

The study was approved by the ethical committee of University of Veterinary and Animal Sciences (Ref. No. IPS/2018/11). Strict patient data confidentiality and compliance with the Declaration of Helsinki were observed.

### 2.3. Data Analysis

Baseline demographic data were presented using descriptive statistics with frequencies and percentages. Data analysis was performed using Microsoft Excel and SPSS (version 23.0). The distribution for each variable in all categories was examined by an analysis of frequencies and percentages.

## 3. Results

**Figure 1:**
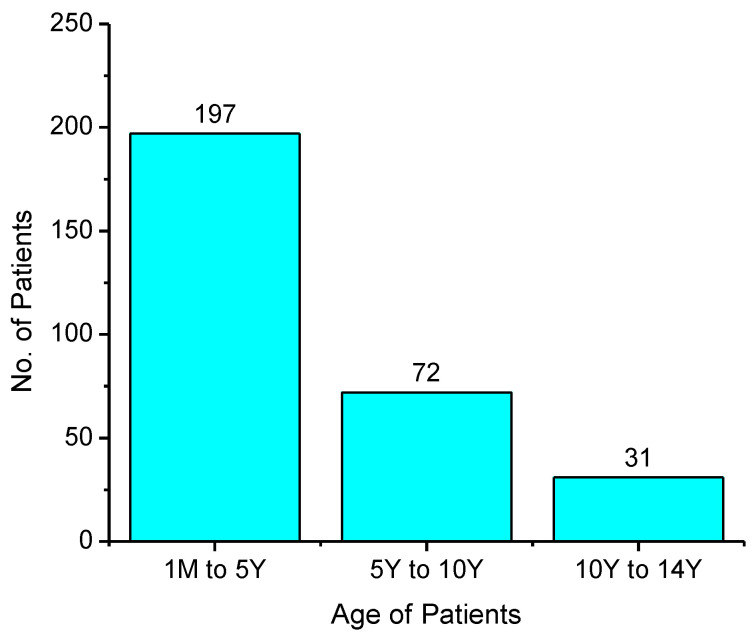
Occurrence of types of DDIs in examined prescriptions.

Data were collected from 300 hospitalized patient files that belonged to four different wards including medical, neurology, nephrology and cardiac wards of a tertiary care hospital. Of the patients, 38.6% were males, and 61.4% were females. The patients belonged to different age groups ranging from 1 month to 5 years (65.67%), 5.1 to 10 years (24%) and 10.1 to 14 years (10.34%). All patients were below 14 years of age (Figure 1).

A total of 434 drug interactions were found in the medical records using Medscape drug interaction checker and categorized according to severity level, i.e., severe, moderate and minor [14]. Most of the drug interactions were considered moderate (58.1%, 252/434), followed by minor (29.9%, 130/434) and severe (11.9%, 52/434). These interactions included different kinds of drug pairs as shown in Table 1. The highest percentage of DDIs was found to be for ceftriaxone and calcium gluconate combination (25%, severe). Ceftriaxone co-administration with phenytoin stood second in prevalence (13.5%, minor). It is worth mentioning that most of the drugs that showed DDIs belonged to anti-infective group, e.g., ceftriaxone, amikacin, vancomycin, ciprofloxacin, clarithromycin, moxifloxacin, clarithromycin and rifampicin. Anti-hypertensive agents including captopril (9.60%), losartan (5.80%), furosemide (3.80%), spironolactone (1.90%), etc. also showed greater numbers of DDIs. The remaining drugs that showed higher percentages of DDIs were analgesics, e.g., piroxicam and tramadol (Table 1).

**Figure 2:**
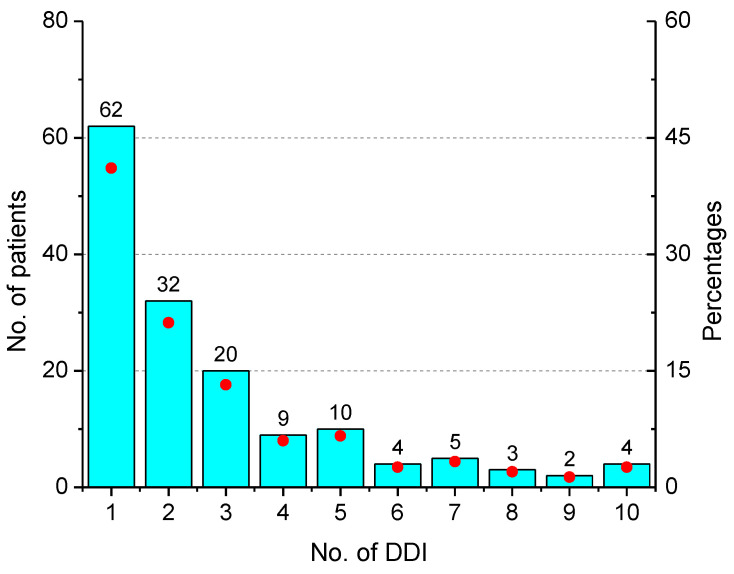
Distribution of patients/prescription orders on the basis of number of drug interactions reported (*n* = 157).

After screening the patients’ medical records, it was found that more than half (52.3%, 157/300) had experienced DDIs, and more than 40% of those had only one drug interaction (Figure 2). As expected, the number of DDIs increased with an increase in the number of prescribed drugs. There were 32 prescriptions with one to three prescribed drugs, while the remainder had more drugs in each prescription.

## 4. Discussion

In the current study, 300 prescriptions were analyzed from pediatric patients; almost half (52.3%, 157/300) of the prescriptions were found to have caused drug-drug interactions. Most of the interactions were found to be moderate (58.1%); however, minor (29.9%) and severe (11.9%) interactions were also found. Pharmacodynamics-based interactions were prevalent compared to interactions based on drug pharmacokinetics. Most of the prescriptions (89.34%) included more than three drugs prescribed, and only 10.66% included 1–3 drugs per prescription. Polypharmacy and prospective drug-drug interactions have been a therapeutic challenge among inpatients [15]. Such investigations among pediatric inpatients have been lacking; therefore, the available data are sparse. In this context, our study was designed to analyze the prospective drug-drug interactions among pediatric patients in a tertiary care hospital. Reducing the polypharmacy, finding non-pharmacological possibilities, re-assessing the treatment options routinely, modifying dosage schedules, adjusting dosage and continuous monitoring for signs of effectiveness and toxicity could be possible approaches for minimizing DDIs.

The most frequent drug-drug interaction (25%, ceftriaxone and calcium gluconate) (Table 1) was categorized as severe and of the pharmacokinetic type. This interaction has also been previously reported in both pediatric and adult patients [16,17]. Co-administration of these drugs can lead to particulate precipitation with risk of end-organ damage. It has been recommended that comixing of calcium-containing IV solutions and ceftriaxone should be avoided to prevent the precipitation reactions. The second most prevalent interaction (13.5%, ceftriaxone with phenytoin) (Table 1) was categorized as minor and of the pharmacodynamic type. The concomitant therapy can displace phenytoin from conventional protein carriers in vitro and in vivo. This interaction has also been reported before [18,19] and could lead to increased serum phenytoin concentrations. It has been recommended to monitor free phenytoin concentration to avoid any toxic reactions in patients, especially in hypo-albuminemic situations [18].

Captopril co-administration with other drugs was found to be highly prevalent. Most drug-drug interactions were of the pharmacodynamic type and of moderate to severe levels (Table 1). Its interaction with piroxicam (9.60%), ciprofloxacin (7.70%) and losartan (5.80%) can interfere with the metabolism of other drugs and can lead to concentration-based toxicity events in these patients. Toxic piroxicam levels could result in gastro-intestinal symptoms, electrolyte imbalance, seizure, coagulopathy and bone marrow aplasia in pediatric patients [20]. Concurrent use of ciprofloxacin with captopril can lead to elevated serum ciprofloxacin levels and results in musculoskeletal (arthralgia) adverse events in children [21]. Toxic concentrations of losartan, due to coadministration with captopril, can also lead to pharmacodynamic synergism and unwanted events in both children and adults.

Anti-infective agents were identified as the most frequently prescribed drug-combination categories (Table 1, Sr. No. 3, 7, 10, 16 and 17). Most of these co-administrations could lead to severe drug-drug interactions and result in unwanted toxic events in the patients. The reason for pairing one anti-infective agent with another, similar drug could be the desire to achieve enhanced efficacy in patients with severe morbid conditions [14]. However, these drug interactions can result in unwanted toxic effects in critically ill patients, especially in children. Strong monitoring for dose adjustments and adverse effects and identifying possibilities for alternative therapies are recommended in these patients.

These facts and figures show that drug-drug interactions are a challenging problem in health care systems. There are several factors that contribute to the occurrence of potential drug-drug interactions in tertiary care settings. These include polypharmacy, non-availability of clinical pharmacists, and poor hospital and government policies regarding drug-drug interactions. Polypharmacy is one of the major reasons for DDIs in secondary and tertiary health care settings [15]. Decreasing the number of drugs per prescription can effectively lessen the incidence of DDIs [22]. However, sometimes in chronically ill patients with cardiovascular disorders or diabetes, polypharmacy cannot be avoided [23]. These interactions can be minimized when the prescribers and pharmacists have proper knowledge of the modes of action, pharmacogenomics, and pharmacokinetics, including absorption, elimination and metabolism of drugs as well as the clinical expertise to predict the risk factors. Hospital and community pharmacists can play a critical role in preventing DDIs by thoroughly reviewing the prescriptions at the hospital as well as community level. This can be achieved by designing proper screening systems and effective pharmaceutical care plans and individualizing the therapy for patients [24].

The current study does not depict the whole population; only a representative pediatric sample from one city in Pakistan was utilized. Similar studies can be planned in other cities belonging to different provinces of Pakistan in the future.

## 5. Conclusions

Our investigations provided preliminary data on the possibility of drug-drug interactions in pediatric inpatients in a tertiary health care hospital. They also identified highly prevalent drugs and drug categories that are mostly paired in prescriptions and can lead to unwanted adverse effects in patients. A future possible area of study could be drug-disease interactions in patients. Proper education, training, and a system of prescription monitoring can help in lessening such unwanted cases. That goal also demands the development of policies to prevent such events in future and better deal with the situation.

## Figures and Tables

**Table 1:** Reported drug interaction pairs.

Sr. No.	Interaction	Interaction Severity	Clinical Outcomes	Class of Interaction	Prevalence
1	Ceftriaxone + calcium gluconate	Severe	Fatal particulate precipitation in lungs and kidneys.	Pharmacokinetic	25%
2	Ceftriaxone + phenytoin	Minor	Ceftriaxone increases toxicity of phenytoin.	Pharmacodynamic	13.50%
3	Amikacin + vancomycin	Moderate	Both increase nephrotoxicity.	Pharmacodynamic	9.60%
4	Piroxicam + captopril	Moderate	Pharmacodynamic antagonism increases toxicity.	Pharmacodynamic	9.60%
5	Captopril + ciprofloxacin	Moderate	Captopril increases toxicity of ciprofloxacin.	Pharmacodynamic	7.70%
6	Losartan + captopril	Severe	Increases toxicity by pharmacodynamic synergism.	Pharmacodynamic	5.80%
7	Piperacillin + vancomycin	Severe	Increases nephrotoxicity and ototoxicity.	Pharmacodynamic	3.80%
8	Furosemide + amikacin	Severe	Increase toxicity by pharmacodynamic synergism.	Pharmacodynamic	3.80%
9	Sodium bicarbonate + digoxin	Severe	Sodium bicarbonate enhances the digoxin levels by increasing gastric pH.	Pharmacokinetic	3.80%
10	Clarithromycin + moxifloxacin	Severe	Clarithromycin and moxifloxacin both increase QTc interval.	Pharmacodynamic	3.80%
11	Aspirin + spiranolactone	Moderate	Aspirin decreases effect of spironolactone.	Pharmacodynamic	1.90%
12	Carbamazepine + nitrazipam	Moderate	Carbamazepine affects the hepatic/intestinal enzyme CYP3A4 metabolism and decreases the level or effect of diazepam.	Pharmacokinetic	1.90%
13	Linezolid + tramadol	Severe	Both increase serotonin level. Linezolid increases serotonin by inhibition of MAO.	Pharmacodynamic	1.90%
14	Promethazine + clarithromycin	Severe	Both increase QT interval.	Pharmacodynamic	1.90%
15	Clarithromycin + hydrocortisone	Severe	Clarithromycin affects the hepatic/intestinal enzyme CYP3A4 metabolism and enhances the hydrocortisone level/effect.	Pharmacokinetic	1.90%
16	Rifampicin + isoniazid	Severe	Rifampicin increases toxicity of isoniazid.	Pharmacokinetic	1.90%
17	Rifampicin + pyrazinamide	Severe	Rifampicin increases toxicity of pyrazinamide.	Pharmacodynamic	1.90%

## Data Availability

The data presented in this study are available on request from the corresponding author.
